# miR-223 Is a Coordinator of Breast Cancer Progression as Revealed by Bioinformatics Predictions

**DOI:** 10.1371/journal.pone.0084859

**Published:** 2014-01-06

**Authors:** Eva Maria Pinatel, Francesca Orso, Elisa Penna, Daniela Cimino, Angela Rita Elia, Paola Circosta, Patrizia Dentelli, Maria Felice Brizzi, Paolo Provero, Daniela Taverna

**Affiliations:** 1 Molecular Biotechnology Center (MBC), University of Torino, Torino, Italy; 2 Department of Molecular Biotechnology and Health Sciences, University of Torino, Torino, Italy; 3 Center for Molecular Systems Biology, University of Torino, Torino, Italy; 4 Department of Medical Sciences, University of Torino, Torino, Italy; 5 Center for Translational Genomics and Bioinformatics, San Raffaele Scientific Institute, Milan, Italy; Sudbury Regional Hospital, Canada

## Abstract

MicroRNAs are single-stranded non-coding RNAs that simultaneously down-modulate the expression of multiple genes post-transcriptionally by binding to the 3′UTRs of target mRNAs. Here we used computational methods to predict microRNAs relevant in breast cancer progression. Specifically, we applied different microRNA target prediction algorithms to various groups of differentially expressed protein-coding genes obtained from four breast cancer datasets. Six potential candidates were identified, among them miR-223, previously described to be highly expressed in the tumor microenvironment and known to be actively transferred into breast cancer cells. To investigate the function of miR-223 in tumorigenesis and to define its molecular mechanism, we overexpressed miR-223 in breast cancer cells in a transient or stable manner. Alternatively we overexpressed miR-223 in mouse embryonic fibroblasts or HEK293 cells and used their conditioned medium to treat tumor cells. With both approaches, we obtained elevated levels of miR-223 in tumor cells and observed decreased migration, increased cell death in anoikis conditions and augmented sensitivity to chemotherapy but no effect on adhesion and proliferation. The analysis of miR-223 predicted targets revealed enrichment in cell death and survival-related genes and in pathways frequently altered in breast cancer. Among these genes, we showed that protein levels for STAT5A, ITGA3 and NRAS were modulated by miR-223. In addition, we proved that STAT5A is a direct miR-223 target and highlighted a possible correlation between miR-223 and STAT5A in migration and chemotherapy response. Our investigation revealed that a computational analysis of cancer gene expression datasets can be a relevant tool to identify microRNAs involved in cancer progression and that miR-223 has a prominent role in breast malignancy that could potentially be exploited therapeutically.

## Introduction

Breast cancer is the tumor with the highest incidence in women [1]. However, recently, life expectancy improved enormously, thanks to early detection, better characterization of tumor molecular parameters and more accurate therapy [2]. Breast cancer is a heterogeneous group of neoplasms derived from the epithelium surrounding the milk ducts [3]. This heterogeneity led to pathology-driven classifications, recently complemented by molecular characterizations. In fact, based on protein-coding gene expression profiling, breast tumors can be classified at least in three major subtypes: luminal or estrogen receptor positive (ER+), basal or triple negative and human epidermal growth factor receptor 2 positive (HER2+) breast tumors [4], [5], [6], [7], which are associated with different clinical outcome. This classification helps in addressing clinical treatment, but the identification of patients that are prone to recur or to develop therapy resistance is far from being achieved. Recently, new tumor features such as tumor-stroma composition [8], [9], [10], [11], [12] and microRNA expression in tumors or stroma cells were shown to be relevant for breast cancer progression and they deserve deep investigation [13], [14], [15], [16], [17].

Stroma composition and tumor-stroma interaction and co-evolution have been found to mediate cancer progression based on chemokine and hormone secretion [18], as well as on exosome or microvesicle production [19], [20].

MicroRNAs are small endogenous non-coding RNAs able to post-transcriptionally downregulate expression of multiple specific target genes by binding to the 3′ UTRs of their mRNAs causing destabilization, degradation or translation inhibition [21]. Several microRNAs, were found to control breast cancer tumor formation and progression, functioning as oncomiRs or tumor suppressor miRs or metastamiRs. Examples are miR-21, miR-155, miR-10b, miR-373, miR-206, miR-17-5p, miR-200 family, let7, miR-34 and miR-31 [22], [23], [24], [25], [26], [27], [28], [29], [30], [31]. MicroRNA expression profiling is of great help for tumor classification since they seem to classify tumors more precisely than protein-coding genes, according to lineage and differentiation status [32], [33]. MicroRNAs can also represent a relevant link between tumor and stroma cells. In fact, microRNAs are often present in exosomes or microvesicles [34], [35] produced by stroma cells and transferred into tumor cells, affecting malignancy. As a consequence, it is particularly important to identify microRNAs involved in tumor-stroma co-evolution.

The focus of our work was the identification of microRNAs, produced by tumor or stroma cells, involved in breast cancer malignancy using *a target reverse gene expression approach* starting from breast cancer gene expression datasets. This approach unravelled a group of six microRNAs, miR-19ab, miR-200bc, miR-203, miR-21, miR-223 and miR-340, predicted to be deregulated during breast cancer progression. Among them, we studied the function and the molecular mechanism of miR-223 in breast cancer malignancy.

## Materials and Methods

### Human Breast Cancer Datasets

Four datasets were used for differential protein-coding gene expression analysis and microRNA prediction: van de Vijver-NKI (http://bioinformatics.nki.nl/data.php) containing expression of 295 consecutive breast tumors, not treated with adjuvant therapy [36]; Pawitan-Gene Expression Omnibus (GEO) series GSE1456 (http://www.ncbi.nlm.nih.gov/geo/query/acc.cgi?acc=GSE1456), 159 patients [37] and Miller datasets, GEO series GSE3494 (http://www.ncbi.nlm.nih.gov/geo/query/acc.cgi?acc=GSE3494), 251 patients [38]: in these two cases tumor selection was done on consecutive samples based on RNA quality and tumor tissue quantity; Desmedt, GEO series GSE7390 (http://www.ncbi.nlm.nih.gov/geo/query/acc.cgi?acc=GSE7390), 198 samples of lymph-node negative patients [39].

### Differential Expression Analysis

The gene expression datasets listed above were normalized using RMA as implemented in the affy package [40] of Bioconductor [41]. Only probes unambiguously linked to unique gene IDs were evaluated. When multiple probes annotated to the same gene were present only the probe having the highest median expression value was considered. For Affymetrix platforms we used manufacturer-provided annotation, version 30, while for NKI dataset we considered the annotation file present on the website. The correspondence to Entrez and Ensemble gene IDs was obtained from BioMart or Entrez gene ftp site. Genes having a p-value lower than 0.05 after Wilcoxon rank-sum and Benjamini-Hochberg correction for multiple testing were used to obtain separate lists of up-regulated and down-modulated genes according to 5 years disease free survival (DFS) status for each dataset. The analysis was performed independently for Entrez and Ensembl gene ID annotated probes to avoid a bias in the next steps, since the results of each prediction algorithms were given in terms of one of these two gene annotation systems.

### microRNA Prediction Analysis

TargetScan, release 5.0, Miranda, release September 2008, MicroCosm (miRBase) Targets v5 and DIANA-microT v3.0 prediction algorithms were used to identify predicted microRNA targets [42], [43], [44], [45]; for all predictions and microRNA nomenclature we referred to miRBase v13. To evaluate enrichments in microRNA seeds, among the differentially expressed gene lists, we used an exact Fisher test. We adjusted the p-values for multiple testing with the Benjamini-Hochberg correction and filtered out the results having a corrected p-value lower than the one corresponding to the 0.99 percentile of the p-value distribution, obtained by randomizing the association between microRNAs and target genes. The highest nominal p-value considered after randomization was between 0.0054 and 0.0078 depending on the prediction algorithm used. Only microRNAs predicted and effectively expressed in breast cancer samples according to Cimino et al. [46] dataset were considered for enrichment analysis.

When we compared our results with what obtained using the method published in [47], t-value was calculated for all the genes present in at least 60% of the samples, according to presence or absence, as evaluated in the PanP Package (http://www.bioconductor.org/packages/2.12/bioc/html/panp.html). Then, a Kolmogorov–Smirnov test was performed and p-values lower then 0.05 (after the Benjamini-Hochberg correction) were considered significant to identify relevant predictions for the previously identified microRNAs (six). All the analyses were performed using R language [48].

### Cell Culture

HEK293, MDAMB231, MCF7 and HeLa cells were obtained from American Type Culture Collection (ATCC); Mouse Embryo Fibroblasts (MEFs) were derived from C57/B6 E13.5 mouse embryos; all maintained in Dulbecco’s Modified Eagle’s Medium (DMEM) containing 10 mM Glutamax and 4.5 g/L glucose (DMEM Glutamax™, GIBCO Invitrogen Life Technologies, Carlsbad, CA), supplemented with 10% heat-inactivated FCS (Biochrom AG, Berlin, DE), 1 mM sodium pyruvate, 25 mM HEPES pH 7.4 and 100 µg/mL gentamycin (all from GIBCO Invitrogen Life Technologies, Carlsbad, CA). T47D were obtained from ATCC and maintained in Roswell Park Memorial Institute (RPMI) medium enriched as described above for DMEM medium plus 5 µg/mL insulin. SUM149PT cells were a gift of Prof. SP Ethier and were cultured as described in [49]. In experiments in which Conditioned Medium (CM) was used, MDAMB231 cells were grown, at different time points, in CM collected from MEFs (P3) or HEK293 cells stably transduced (or not) with pLemiR-empty (empty) or pLemiR-miR-223 (miR-223) expressing lentiviral vectors. For all biological assays in which we used HEK293 cells CM medium on MDAMB231 cells, starvation for 3 days was performed in HEK293 cell cultures.

### Human Breast Tumor Samples

Paraffin embedded tumor specimens were selected from the Tumor Bank of the Department of Obstetrics and Gynecology, University of Turin, obtained from patients who underwent primary surgical treatment. Appropriate ethical approval was obtained for this study [46]. These samples were used to extract RNA via punches as indicated below.

### Reagents, Antibodies and Primers

**RNAi:** si-STAT5A (Hs_STAT5A_5) and si-control (AllStars Negative Controls) (from QIAGEN Stanford, CA). **microRNA precursors**: Pre-miR™ microRNA Precursor Molecules for Negative Control#1, Hsa-miR-223 (PM12301) or Hsa-miR-203 (PM10152) and Hsa-miR-196 (PM10068) used as unrelated microRNAs (controls for some experiments). **microRNA detection**: TaqMan® MicroRNA Assays for Hsa-miR-19a (ID 000395), Hsa-miR-200b (ID 002251), Hsa-miR-203 (ID 000507), Hsa-miR-21 (ID 000524), Hsa-miR-223 (ID 002295), Hsa-miR-340 (ID 000550), U6 snRNA (ID 001973) (all from Applied Biosystems, Foster City, CA). **Primary antibodies**: anti-STAT5A L-20, anti-N-RAS mAb F155, anti-GAPDH Ab V-18 (from Santa Cruz Biotechnology, Santa Cruz, CA), and anti-α-TUBULIN mAb B5-1-2 (from Sigma, St Louis, MO), anti-VINCULIN kindly provided by G. Tarone (Molecular Biotechnology Center, University of Torino, Italy), anti-ITGA3 pAb 8-4 B7 gently provided by Mike DiPersio [50]. **Secondary antibodies**: goat anti-mouse IgG HRP-conjugated, goat anti-rabbit IgG HRP-conjugated, donkey anti-goat IgG HRP-conjugated (all from Santa Cruz Biotechnology, Santa Cruz, CA). All antibodies were used at the producer’s suggested concentrations. **Adhesion:** Collagen IV, Fibronectin, Laminin from Sigma Aldrich, St Louis, MO. **Cell death reagents:** FITC-conjugated Annexin V and PI were from Bender MedSystems (Vienna, Austria). APC-conjugated Annexin V was from BD Biosciences (Bedford, MA). Tetramethylrhodamine methyl ester (TMRM) was from Molecular Probes (Invitrogen, Carlsbad, CA). Paclitaxel (PTX) was an ONCOTAIN trademark (MaynePharma, AU) and Doxorubicin was from Sigma Chemical Co. (St Louis, MO). Z-VAD-FMK was from Promega (Madison, WI). **Primers:** miR-223 fw: ccgctcgagGAGCTTCCAGCTGAGCACTGGG; miR-223 rev: cgacgcgtTATTGCGCCCCCATCAGCACT; Stat5a fw: aaactagtTTGACTCCCGCCTCTCGCCC; Stat5a rev: ttacgcgtCCTCTTCTCATCCCCACCTCCCT; Stat5a mut sense: tttagtaaggctgtgtacacgggcccctttgcaggcatgcatgtg; Stat5a mut antisense: cacatgcatgcctgcaaaggggcccgtgtacacagccttcataaa.

### Vector Construction and Lentiviral Infections

The 3′UTR of STAT5A, previously generated by PCR amplification of the full length 3′UTR from human cDNA of MDAMB231 cells was inserted in the pMIR REPORT™ luciferase vector (Ambion, Austin, TX) generating the STAT5A vector. miR-223 binding site in the 3′UTR was mutagenized (STAT5AMUT) using the QuickChange Site-Directed Mutagenesis kit (Stratagene, Cedar Creek, TX) according to the manufacturer’s instructions.

The human STAT5A cDNA, containing the full length 3′ UTR, was kindly obtained from Prof. B. Groner (Goethe University of Frankfurt am Main, Germany) and cloned into the pCMV-EGFP vector after GFP removal. miR-223 binding site on STAT5A 3′UTR was then mutagenized as described for pMIR REPORT-luciferase-STAT5A.

The human pre-miR-223sequence (a 559 bp fragment containing the premiR sequence) was amplified from genomic DNA (MDAMB231) and cloned into pLemiR-tRFP (Open Biosystems, Huntsville, AL) vector to obtain pLemiR-223 (still containing tRFP) vector. Lentiviruses were produced by calcium phosphate transfection of 293 T cells with 20 µg of specific vector together with 15 µg packaging (pCMVdR8.74) and 6 µg envelope (pMD2.G-VSVG) plasmids according to Trono’s lab protocol (http://tronolab.epfl.ch). Supernatant was harvested 48 h post-transfection, filtered with 0.45 µm filters and used to infect 3.5×10^5^ cells in 6-well plates, in presence of 8 µg/mL Polybrene (Sigma-Aldrich, StLouis, MO).

### Transient Transfections of Pre-microRNAs and siRNAs

To obtain transient pre-miR, or siRNA expression, cells were plated in 6 or 12 well plates at 50–70% confluency and transfected using HiPerFect Transfection Reagent (QIAGEN, Stanford, CA) reagent, according to manufacturer’s instructions, with 75 nM of pre-miR or 100 nM siRNA. Cells were tested for microRNA or protein-coding gene overexpression/knockdown 48 h later. For transient cDNA overexpression, cells were plated at 90% confluency and transfected 24 h later using Lipofectamine 2000™ reagent (Invitrogen Life Technologies, Carlsbad, CA).

### RNA Isolation and qRT-PCR for microRNA or mRNA Detection

Total RNA was isolated from cells using TRIzol® Reagent (Invitrogen Life Technologies, Carlsbad, CA) according to manufacturer’s protocol. Instead, RNA from formalin-fixed paraffin embedded breast tumor specimen punches was obtained as follows. Microscopical slides of paraffin inclusions were scanned with Panoramic Desk (3DHistech, Euroclone, Pero, MI, Italy) and corresponding virtual slides were evaluated with the Panoramic View program (3DHistech). From each tumor 2 areas of sampling (1 mm in diameter) were marked on the virtual slides. Virtual slides with sampling markers were transferred to the TMA instrumentation (Panoramic Desk, 3DHistech). Low magnification images of the slides were matched with the corresponding histological block inclusions and the selected areas were punched out with a 1 mm punching needle. From each block 1 mm cores were collected in custom vials, inserted in the waste bin receptacle, properly labeled and RNA isolated using acid guanidinium thiocyanate-phenol-chloroform extraction method. All RNA quantitations were performed using the NanoDrop-1000 spectrophotometer (Nanodrop, Wilmington, DE). qRT-PCRs for detection were performed with the indicated TaqMan® MicroRNA Assays (Applied Biosystems, Foster City, CA) on 10 ng total RNA according to the manufacturer’s instructions. Quantitative normalization was performed on the expression of the U6snoRNA. The relative expression levels between samples were calculated using the comparative delta CT (threshold cycle number) method (2^−ΔΔ*C*T^) with a control sample as reference point [51]. RNA samples from monocytes (CD14), dendritic cells (DC, TNFα-activated DC), activated T-cells (anti-CD3/CD28), purified T-cells (CD8), hematopoietic stem cells (CD34) and mesenchymal cells (MSC) were kindly provided by A. Cignetti (MBC, Torino, Italy).

### Migration and Invasion Transwell Assays

To measure migration 8×10^4^ MDAMB231 were seeded in serum-free media in the upper chambers of cell culture inserts (transwells) with 8.0 µm pore size membrane (24-well format, Becton Dickinson, NJ). Invasion assays were performed using BioCoat^TM^Matrigel Invasion Chambers with 8.0 µm pore size membrane (Becton Dickinson, NJ). For migration and invasion the lower chambers were filled with complete growth media. After 20–24 h, the migrated cells present on the lower side of the membrane were fixed in 2.5% glutaraldehyde, stained with 0.1% crystal violet and photographed using an Olympus IX70 microscope. Migration and invasion were evaluated by measuring the area occupied by migrated cells using the ImageJ software (http://rsbweb.nih.gov/ij/) [52].

### Adhesion Assays

To test adhesion, 5×10^4^ cells/well were seeded directly on 5 µg/mL collagen IV or 10 µg/mL fibronectin or 5 µg/mL laminin (all from Sigma-Aldrich, St Louis, MO) precoated 96-well plates, for 1 h at 37°C. Cells were then washed thoroughly to remove non adherent cells, fixed with methanol and stained with haematoxylin and eosin (Diff-Quik, Medion Diagnostics, Dudingen, CH). Wells were photographed using Olympus IX70 microscope and the area occupied by the adherent cell was measured by using the ImageJ software (http://rsbweb.nih.gov/ij/) [53].

### Proliferation Assays

5×10^3^ cells/well were plated in 96-well plates in complete medium and starved for 12–24 h. Complete medium was then added and cells were allowed to grow for 24, 48, 72, 96 hours, fixed with 2.5% glutaraldehyde and stained with 0.1% crystal violet. The dye was solubilised using 10% acetic acid and optical density measured directly in plates using GloMax Luminometer (Promega, Madison, WI) at 570 nm wavelength [54].

### *Anoikis* Assay

Cells were plated on a 2% agarose pad in serum-free medium for 48 h, collected, washed in PBS buffer, resuspended in 10 mM Hepes, 150 mM NaCl, 5 mM CaCl_2_ buffer containing FITC-conjugated Annexin-V (Bender MedSystems, GmbH) and 200 nM tetramethyl-rhodamine-methyl-ester (TMRM, Molecular Probes, Invitrogen, CA) and incubated at 37°C for 20 minutes. Flow cytometry analysis of *anoikis* was carried out using a FACSCalibur flow cytometer (Becton Dickinson, NJ). Data acquisition was performed using CellQuest software (Becton Dickinson, NJ) and data analysis with WinMDI software (version 2.8, Scripps Institute, CA). Results were displayed in bidimensional plots, with gates indicating the percentages of healthy and dead cell populations [55].

### Cell Death Assays

1–1.5×10^5^ MDAMB231 were plated in 12 well plates and transfected as already described, 24 h after cells were washed and grown in complete medium with or without 1 µM Paclitaxel (PTX) or 1 µM Doxorubicin (DOXO) for 48 h. When present, Z-VAD-FMK inhibitor was used at 20 µM final concentration in the presence of 1 µM PTX. The supernatant was collected and cells were washed once in phosphate-buffered saline (PBS) detached by trypsinization and added to the supernatant suspension. Labeling and analysis was performed as in *anoikis* analysis.

### Luciferase Assays

6.5×10^4^ cells were cotransfected with 50 ng of the pMIR REPORT™ (Ambion, Austin, TX) Firefly Luciferase constructs containing the 3′UTRs of the indicated miR-223 potential target, 20 ng of pRL-TK Renilla Luciferase normalization control (Promega, Madison, WI) and 75 nM of the indicated pre-miR using Lipofectamine^TM^2000 (Invitrogen Life Technologies, Carlsbad CA). Lysates were collected 48 h after transfection and Firefly and Renilla Luciferase activities were measured with a Dual-Luciferase Reporter System (Promega, Madison, WI).

### Protein Preparation and Immunoblotting

Total protein extracts were obtained using a boiling buffer containing 0.125 M Tris/HCl, pH 6.8 and 2.5% sodium dodecyl sulphate (SDS). 25 or 50 µg proteins were separated by SDS polyacrylamide gel electrophoresis (PAGE) and electroblotted on to polyvinylidene fluoride (PVDF) membrane Immobilon-P (Millipore, Billerca MA). Membranes were blocked in 5% non-fat milk Phosphate buffered saline PBS-Tween buffer (137 mM NaCl, 2.7 mM KCl, 8 mM Na_2_HPO_4_, 1.46 mM KH_2_PO_4_, 0.1% Tween-20) for 1 h at 37°C, then incubated with appropriate primary and secondary antibodies in 1% milk or BSA (Sigma) PBS-Tween buffer, respectively overnight at 4°C and for 1 h at room temperature and visualized by enhanced chemiluminescence (ECL®, GE Healthcare Life Sciences, GmbH).

### Statistical Analyses of Biological Samples

Unless otherwise noted, data are presented as mean ± Standard Error of the Mean (SEM) and two tailed Student’s t test was used for comparison, with * = p<0.05; ** = p<0.01; *** = p<0.001 considered to be statistically significant. n.s. indicates a not statistically significant p-value.

### Ingenuity Pathway Analysis

Only miR-223 targets predicted by at least two out of 4 prediction algorithms (TargetScan, release 5.2, Miranda, release August 2010, MicroCosm (miRBase) Targets v5 and DIANA-microT v3.0) were considered for Ingenuity Pathway Analysis (IPA) [42], [43], [44], [45]. The Ingenuity Pathways Knowledge Base (http://www.ingenuity.com/) is currently the world’s largest database of knowledge on biological networks, with annotations curated by experts. We exploited this database to look for enrichments in cellular functions, pathways or disease related genes among miR-223 putative targets. Enrichment significance in Signaling pathways analysis is shown as the negative Log10 of the p-value. The p-value is calculated with the right-tailed Fisher’s Exact Test. Ratio is calculated as the number of predicted targets over the total gene number of each pathway.

## Results

### 6 microRNAs are Predicted to be Involved in Breast Cancer Progression

**Figure 1:**
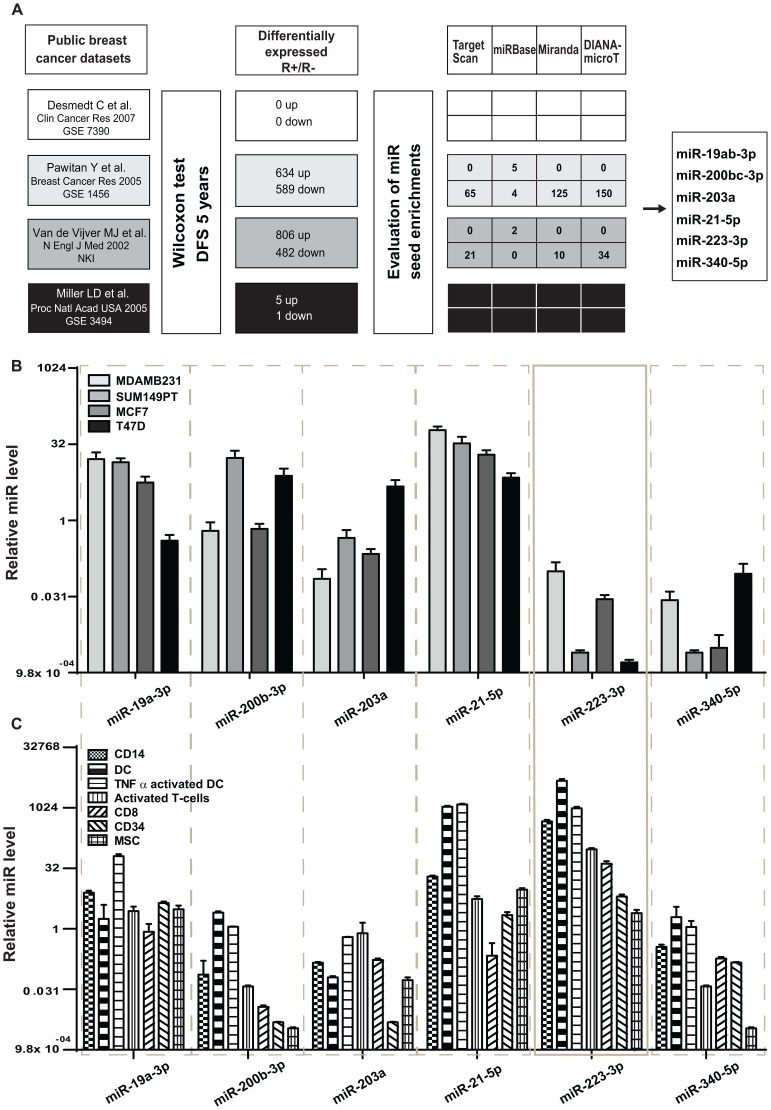
Prediction of miRs involved in breast cancer progression and their expression in cells. (**A**) Four public datasets of primary breast cancers were used to identify differentially expressed genes comparing patients with or without disease relapse, five years post-surgery (DFS = disease free survival). An hypergeometric test was applied to reveal microRNA seed enrichments, according to the predictions provided by at least two algorithms among TargetScan, Miranda, miRBase (MicroCosm Targets) and DIANA-microT and six miRs were identified. (**B–C**) Expression of the six predicted microRNAs in breast cancer cell lines (**B**), such as ER- highly aggressive, MDAMB231 and SUM149PT; ER+ non invasive, MCF7 and T47D, and stroma cells (**C**), such as monocytes (CD14), dendritic cells (DC, TNFα activated DC), activated T-cells (CD3/CD28), purified T-cells (CD8), hematopoietic stem cells (CD34) and mesenchymal cells (MSC). Results are presented in a log2 scale, as fold changes (mean±SD) relative to the delta CT mean of triplicates for each biological sample. Delta CTs were obtained after normalization on U6sno RNA level. SD = standard deviation; CT = threshold cycle number. TNFα = Tumor necrosis factor alpha.

To infer a potential correlation between deregulation of microRNAs and breast cancer progression through the analysis of gene expression data, we set up the pipeline shown in Figure 1A. First, we computed the lists of up and down-modulated genes (kept separated) from four breast cancer public available datasets, comparing patients with (R+) or without (R-) disease relapse within five years from surgery. Second, we used the lists of up or down regulated genes, each one including at least 30 differentially expressed genes (only two datasets led to this requirement, Figure 1A), to predict enrichments in microRNA seeds in mRNA 3′UTRs using four prediction algorithms (TargetScan v 5.0, Miranda September 2008, MicroCosm (miRBase) Targets v5 and DIANA-microT v3.0). Third, we prioritized the predicted microRNAs effectively expressed in breast cancer samples as in [46], obtained from two datasets [36], [37] and by at least two different algorithms. In this way, a group of six microRNAs, miR-19ab, miR-200bc, miR-203, miR-223, miR-21 and miR-340 (as from miRBase v13) or miR-19ab-3p, miR-200bc-3p, miR-203a, miR-223-3p, miR-21-5p and miR-340-5p (as from miRBase v20) was revealed. In parallel, we used the *target reverse gene expression approach* proposed in [47] to verify our microRNA predictions. In this way, we used three datasets [37], [38], [39] for microRNA predictions and, once more, miR-19ab, miR-200bc, miR-203, miR-223, miR-21and miR-340 were predicted (Table 1).

**Table 1:** Six microRNA prediction occurrence in datasets according to [47].

Datasets	hsa-miR-19ab-3p	hsa-miR-200bc-3p	hsa-miR-203a	hsa-miR-21-5p	hsa-miR-223-3p	hsa-miR-340-5p
Desmedt C et al. Clin Cancer Res2007 GSE 7390	–	–	–	x(2)	x(1)	x(2)
Miller LD et al. Proc Natl Acad USA2005 GSE 3494	x(3)	x(3)	x(3)	x(3)	x(3)	x(3)
Pawitan Y et al.Breast Cancer Res2005 GSE 1456	x(3)	x(3)	x(3)	x(3)	x(3)	x(3)

Significant enrichments of our six microRNAs among genes expressed in Recurrent (+) versus Non-Recurrent (−) samples, in the indicated breast cancer datasets, as evaluated by [47]. X = predicted. − = non-predicted. In parenthesis we indicate the number of algorithms able to predict the occurrence.

**Figure 2:**
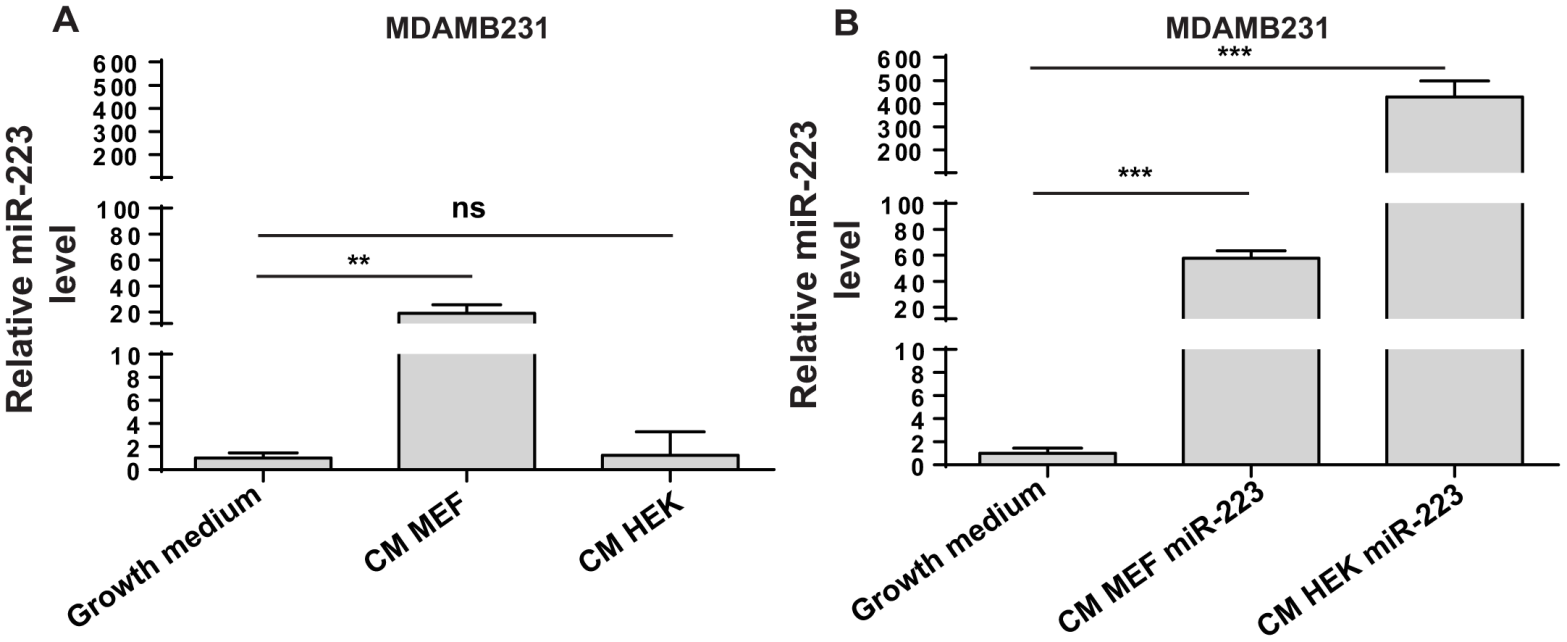
miR-223 expression in MDAMB231 cells grown in Conditioned Medium from miR-223 overexpressing cells. miR-223 levels were measured in MDAMB231 cells grown in normal culture conditions (Growth medium) or in Conditioned Medium (CM) derived from Mouse Embryonic Fibroblasts (MEF) or Human Embryonic Kidney cells (HEK293-HEK) for 48 hours (**A**). Alternatively, CM was derived from the cells in (A) stably transduced with miR-223 overexpression (miR-223) lentiviral vectors (**B**). Results are presented as fold changes (mean±SD) relative to nomal growth conditions. Delta CTs were obtained after normalization on U6sno RNA level. SD = standard deviation; CT = threshold cycle number. Three biological experiments were performed, each with three technical triplicates. Statistics was performed on technical triplicates of one representative biological experiment.*P<0.05; **P<0.01; ***P<0.001.

Expression of these small RNAs was evaluated by qRT-PCR in a panel of human breast cancer cells in culture, including ER−/highly aggressive MDAMB231 and SUM149PT or ER+/poorly aggressive MCF7 and T47D cell lines (Figure 1B, log2 scale). No major differences were evidenced considering malignancy or ER expression. All tumor cells resulted almost empty for miR-223 and miR-340, while showed variable levels for miR-200b and miR-203 and higher levels of miR-19 and miR-21. Considering that tumor-associated stroma cells produce microRNAs that can be transferred into tumor cells through microvesicles and cell-cell junctions, we evaluated the expression of the same microRNAs by qRT-PCR in stroma cells such as monocytes (CD14), dendritic cells treated or not with Tumor Necrosis Factor alpha (DC, TNFα activated DC), activated T-cells (CD3/CD28), purified T-cells (CD8), hematopoietic stem cells (CD34) and mesenchymal stem cells (MSC). While for miR-200b, miR-203 and miR-340 expression was low also in microenvironmental cells, for miR-223 a high expression (10 to 5000 fold increase) was observed in stroma cells compared to breast tumor cells. miR-19a and miR-21 were highly expressed also in some stroma cells, in particular in activated dendritic cells (Figure 1C, log2 scale). Since miR-223 was the only putative small RNA to be expressed uniquely in stroma but not in tumor cells in culture, we hypothesized a possible transfer of miR-223 from stroma to tumor cells within the human tumor mass. To verify our hypothesis, we first evaluated miR-223 expression in pools of RNA derived from tumor or stroma components of human breast tumor samples following dissections (punches) performed in paraffin embedded tumors or stroma as shown in Figure S1A. As shown in Figure S1B, good expression of miR-223 is visible in tumor or stroma components of breast cancer samples as well as in lymph nodes, while MDAMB231 cells are empty. High levels of miR-21, used as a control, were found in all samples and in MDAMB231 cells. In a second approach, we evaluated miR-223 expression in MDAMB231 cells grown for 48 hours in presence of a conditioned medium (CM) derived from mouse embryonic fibroblasts (MEFs) or HEK293 cells or from the same cells previously transduced with miR-223 lentivirus vectors (expression levels in Figure S2A–B). Good/high levels of miR-223 expression were found in MDAMB231 cells when CM from miR-223-overexpressing or control MEFs or HEK293 cells was used, compared to normal growth medium (Figures 2A, B and S2) suggesting a transfer of miR-223 from MEFs or HEK293 cells to MDAMB231 cells. To note that miR-223 was not endogenously expressed by HEK293 cells, while expression was found in MEFs.

### miR-223 Impairs Tumor Cell Migration and Invasion

Given that miR-223 is expressed in stroma cells, that it is expressed in tumor samples and that it could be transferred to breast cancer cells from surrounding cells, we evaluated miR-223 biological functions or target gene expression in MDAMB231 or SUM149PT cells. This was done by overexpressing miR-223 in tumor cells or by growing them in presence of conditioned medium (CM) derived from cells overexpressing miR-223 (see above). When MDAMB231 or SUM149PT cells were stably transduced with miR-223 overexpressing (miR-223) or empty (empty) lentiviral vectors, or transiently transfected with miR-223 precursors or controls (pre-miR-223, pre-control), or grown in miR-223 overexpressing or control HEK293 CM, increased (200 to 10,000 folds) levels of miR-223 were obtained (Figure S2C–F).

**Figure 3:**
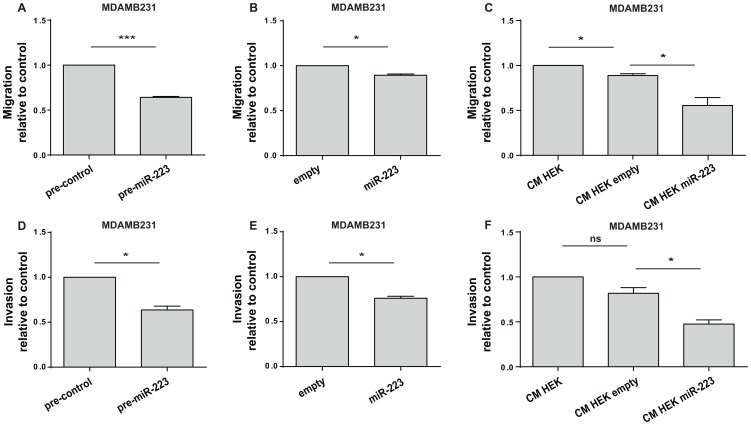
miR-223 reduces cell migration and invasion. Transwell migration (A–B–C) or matrigel invasion (D–E–F) assays. MDAMB231 cells were transfected with miR-223 or their negative controls (pre-miR-223 or pre-control) or stably transduced with pLemiR empty (empty) or miR-223 overexpression (miR-223) vectors or pre-treated for 48 h with conditioned medium (CM) collected from stably transduced HEK293 (HEK) cells with the above mentioned vectors (CM HEK empty or CM HEK miR-223). Results are shown as mean±SEM (standard error mean) of the area covered by migrated cells (A–F). Three independent biological experiments were performed in triplicate (A–F). Triplicate means, normalized on controls, in pools of 3 (A–B–C–D) or 2 (F–G) comparable biological experiments are shown and used for statistics. *P<0.05; **P<0.01; ***P<0.001; ns = not significant.

When MDAMB231 cell proliferation or adhesion on Collagen IV, Fibronectin, Laminin or Plastic was analyzed no differences were observed between overexpressing and control cells (data not shown). Instead a 10% to 40% decrease in cell migration and invasion was found in transwell assays with or without matrigel (Figure 3A–F and S3) in presence of miR-223 overexpression or CM. These findings suggest an anti-invasive function of miR-223 expressed in tumor cells or transferred from surrounding cells.

### miR-223 Expression Enhances Cell Death in Anoikis Conditions or in Presence of Chemotherapeutic Drugs

**Figure 4:**
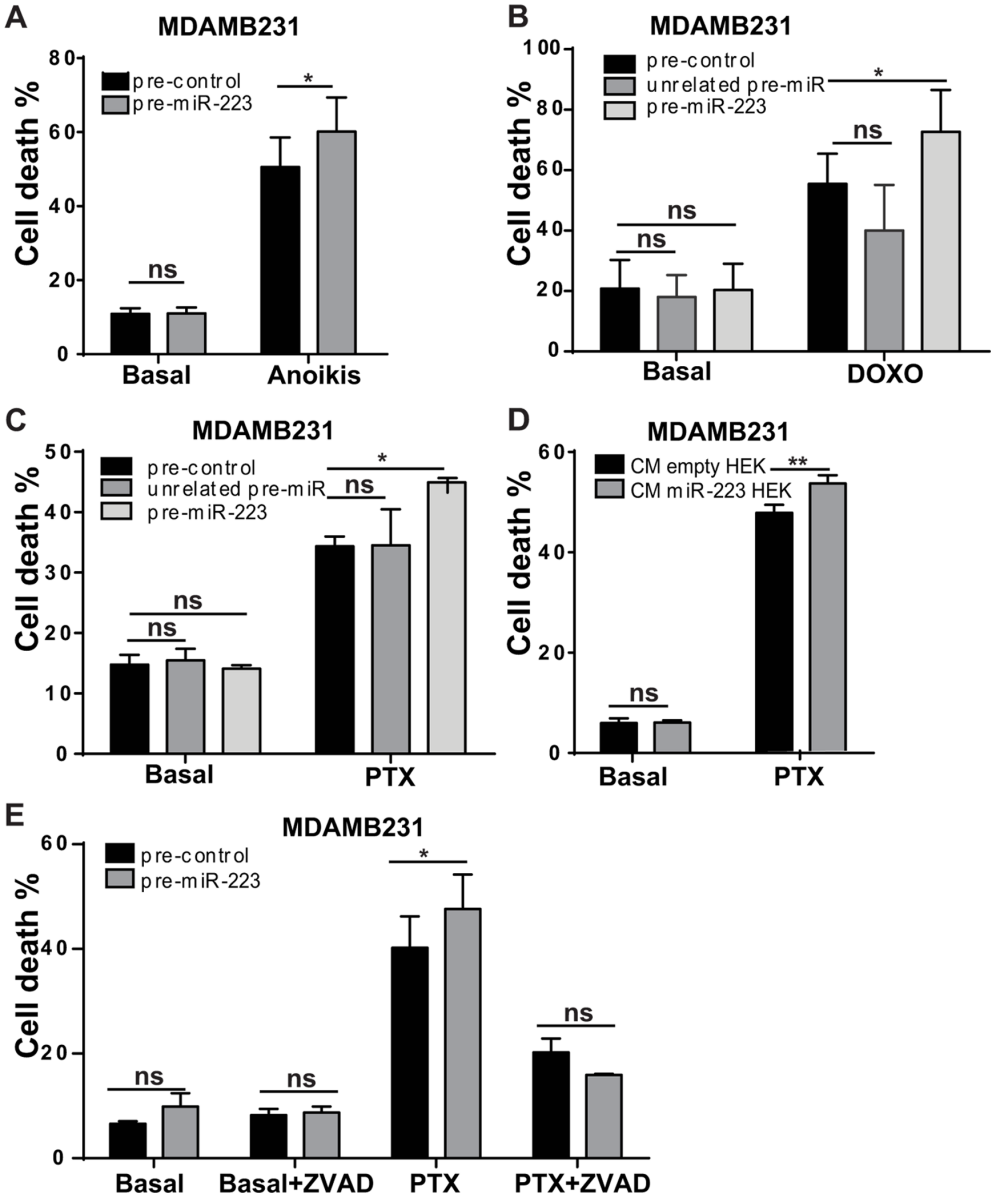
miR-223 enhances anoikis and chemotherapy induced cell death. MDAMB231 cells were grown for 48(**A**) or in complete medium with Doxorubicin (DOXO) (**B**) or Paclitaxel (PTX) (**C**) after transient transfection with miR-223 or with unrelated miR precursors or their negative controls (pre-miR-223 or unrelated pre-miR or pre-control). Alternatively MDAMB231 were grown for 48 h in conditioned medium (CM) collected from HEK293 (HEK) cells stably transduced with pLemiR empty (empty) or miR-223 overexpression (miR-223) vectors. MDAMB231 cells were further transferred to regular medium without (Basal) or with PTX for 48 h and cell death was analyzed (**D**). To control chemotherapy-induced cell death, MDAMB231 cells were treated as in (C) in presence or absence of ZVAD, a caspase inhibitor (**E**). The percentage (%) dead cells displayed in histograms as mean±SEM (standard error mean) was evaluated by TMRM and AnnexinV-FITC or -APC stainings in a FACS analysis. At least three independent biological experiments were performed in duplicate. Duplicate means relative to three or more pooled biological experiments are shown and used for statistics. *P<0.05; **P<0.01; ***P<0.001.

To evaluate the effects of miR-223 up-regulation on MDAMB231 metastatic cell survival in the blood flow, we measured cell viability in absence of anchorage (anoikis) and serum for 48 hours by Annexin V-FITC and TMRM staining in FACS analysis and observed a 10–15% increase of cell death (Figure 4A and S4A). Considering that chemotherapy is the main therapeutic strategy against tumor cells, we investigated the effects of miR-223 overexpression on cell death induced by doxorubicin (DOXO) or paclitaxel (PTX) for 48 hours. Increased cell death was observed in MDAMB231 cells transiently transfected with miR-223 precursors or controls (pre-miR-223, pre-control and unrelated-pre-miR) (Figure 4B, C and S4B, C). Similar results were obtained when MDAMB231 cells were grown for 48 hours in the presence of CM derived from miR-223 overexpressing HEK293 (HEK) cells (Figure 4D and S4D). As a control of cell death, we performed an experiment with MDAMB231 cells transiently transfected with miR-223 precursors or controls (pre-miR-223, pre-control) in which cells were kept or not in presence of PTX and ZVAD, a caspase inhibitor. While no effect on cell death was observed for ZVAD in absence (Basal+ZVAD) of PTX, a cell death inhibitory effect was observed in presence of PTX (PTX+ZVAD), for miR-223 overexpressing cells compared to controls (Figure 4E and S4E, F).

### miR-223 Affects Signal Transduction Pathways Involved in Cell Death and Directly Targets STAT5A

**Figure 5:**
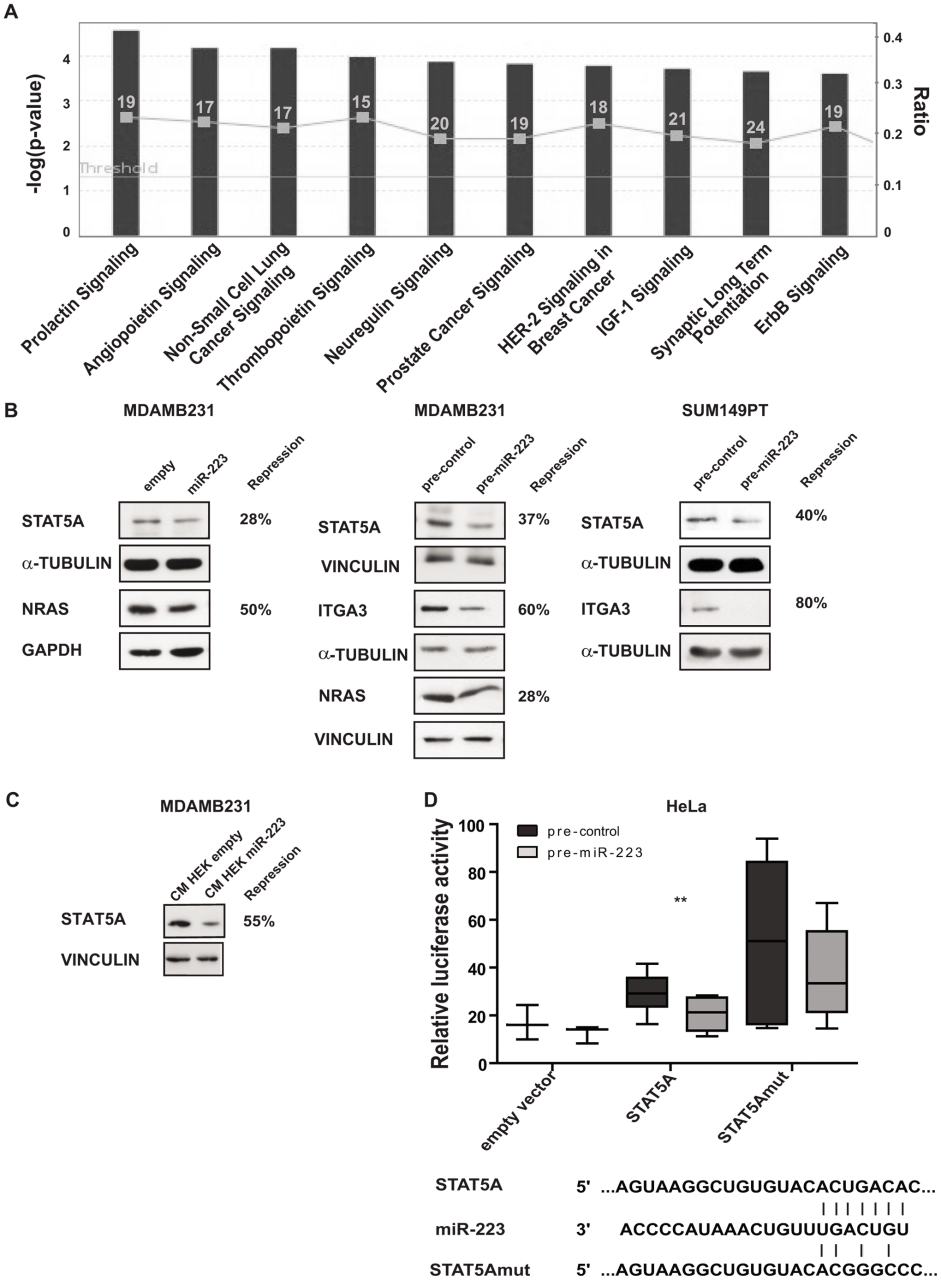
Ingenuity Pathway Analysis of miR-223 predicted targets and STAT5A direct targeting. (**A**) The genes predicted to be miR-223 targets by at least two algorithms among TargetScan, Miranda, miRBase (MicroCosm Targets) and DIANA-MicroT were used to perform an Ingenuity Pathway Analysis (IPA). The top 10 enriched signalling pathways are shown; the dark grey bars represent the -log(p-values) for the members of each pathway (referring to the left Y-axes), the threshold line was set at –log (0.05) for statistical significance. The ratio between miR-223 predicted targets (numbers in each bar) and the total number of genes in each pathway (not shown) is indicated by light–grey squares in each bar (relative to the right Y-axes). (**B–C**) Analysis of STAT5A, NRAS or ITGA3 protein levels by western blot in MDAMB231 or SUM149PT cells stably transduced with pLemiR empty (empty) or miR-223 overexpression (miR-223) vectors or transiently transfected with miR-223 precursors or their negative controls (pre-miR-223 or pre-control) or grown for 48 hours in conditioned medium (CM) collected from HEK293 (HEK) cells stably transduced with pLemiR empty (empty) or miR-223 overexpression (miR-223) vectors. Protein modulations were calculated relative to controls, normalized on α-TUBULIN, GAPDH or VINCULIN as loading controls and expressed as repression percentages. (**D**) Luciferase assays in HeLa cells cotransfected with empty (empty vector) or wild-type (STAT5A) or mutant (STAT5Amut) pMIR-Luciferase reporter vectors, together with miR-223 precursors or negative controls (pre-miR-223 or pre-control). Results are shown as Firefly Luciferase activity normalized on Renilla Luciferase activity. Three to six biological independent experiments were performed, each in triplicate. Triplicate means of each biological experiment are shown as box-plot. *P<0.05; **P<0.01; ***P<0.001. Bottom panel: human miR-223 sequence paired with a portion of the human STAT5A 3′UTR including the wild type or mutant binding site for miR-223.

To identify diseases, functions and pathways controlled by miR-223, we used the pool of 1995 miR-223 predicted targets (predictions by two out of the following four algorithms, TargetScan v5.2, Miranda, August 2010, MicroCosm (miRBase) Targets v5 and DIANA-microT v3) to run an Ingenuity Pathway Analysis (IPA). *Cancer* (582 genes) resulted the top enriched disease while *Cell Death and Survival* (497 genes) was the most enriched cellular function as shown in Table 2. Moreover, an analysis on signaling pathways revealed an enrichment for many pathways including STAT and RAS family members as well as phosphatases and kinases (i.e. PIK3C2A, PIK3R1-3, PTPN11) in the top 10 pathways (Figure 5A). ITGA3, NRAS, STAT5A (all miR-223 predicted targets) protein expression was evaluated in stably or transiently miR-223 overexpressing (miR-223 or pre-miR-223) or control (empty or pre-control) MDAMB231 or SUM149PT cells (Figure 5B). A down-modulation of 60–80%, 28–50% and 28–40% was observed respectively for ITGA3, NRAS and STAT5A in miR-223 overexpressing cells. α-TUBULIN, GAPDH or VINCULIN were used as loading controls. When MDAMB231 cells were treated for 48 hours with conditioned medium (CM) derived from miR-223 overexpressing HEK293 (HEK) cells, decreased levels of STAT5A were observed compared to controls, suggesting a transfer of miR-223 from CM to MDAMB231 cells acting on STAT5A levels. VINCULIN was used as loading control (Figure 5C).

**Table 2:** Diseases and functions related to miR-223 predicted targets.

Disease and disorders		
Name	p-value	# Molecules
Cancer	1.38E-07–6.33E-03	582
Neurological Disorder	6.80E-06–7.00E-03	306
Developmental Disorder	1.02E-05–7.00E-03	182
Gastrointestinal Disease	2.09E-05–6.33E-03	107
Organismal Injury and Abnormalities	2.33E-05–7.00E-03	145
**Molecular and cellular functions**		
**Name**	**p-value**	**# Molecules**
Cell Death and Survival	8.13E-09–6.56E-03	497
Gene Expression	2.12E-08–6.83E-03	357
Cellular Assembly and Organization	3.03E-08–7.00E-03	273
Cellular Function and Maintenance	3.03E-08–6.38E-03	384
Molecular Transport	2.25E-07–6.46E-03	332

N = 1995 targets for miR-223 predicted by at least two algorithms among TargetScan, Miranda, miRBase (MicroCosm Targets) and DIANA-MicroT were used to perform an Ingenuity Pathway Analysis (IPA). The top 5 enriched *Diseases and Disorders* or *Molecular and Cellular Functions* are shown, scored by p-values. The number of predicted miR-223 targets assigned to each group is reported in the last column.

Direct targeting was evaluated on STAT5A 3′UTR in a luciferase assay in miR-223 overexpressing and control (pre-miR-223, pre-control) cells. When HeLa cells were co-transfected with miR-223 and reporter vectors containing the full length wild type (STAT5A) or mutated (STAT5AMUT) 3′UTR or empty vectors, a significant decrease in luciferase activity was specifically observed when miR-223 was overexpressed with wild type but not mutant STAT5A 3′UTR reporter vector, indicating a direct targeting for miR-223 on STAT5A 3′UTR (Figure 5D).

### Down-modulation of STAT5A Accounts for miR-223 Biological Effects

**Figure 6:**
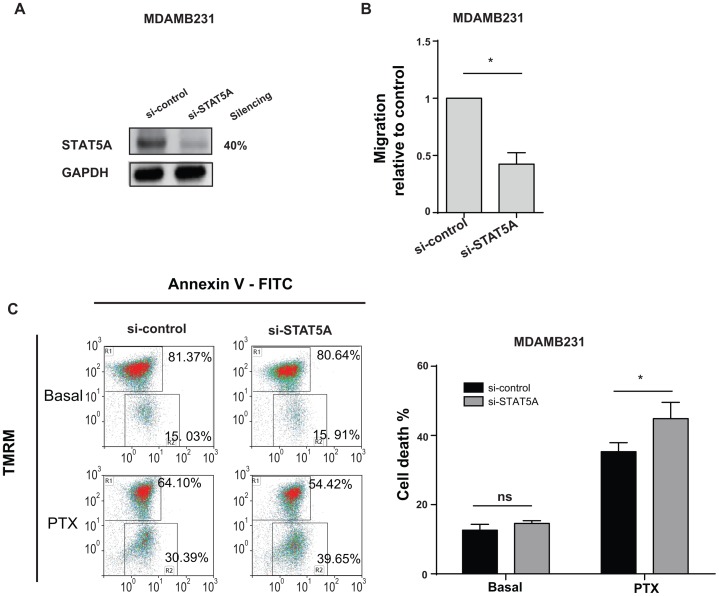
STAT5A downmodulation phenocopies miR-223 functions. MDAMB231 were transiently transfected with STAT5A siRNAs (si-STAT5A) or negative controls (si-control) and protein levels (**A**) or transwell migration (**B**) or cell death induction upon paclitaxel (PTX) treatment (**C**) were evaluated. (**A**) Protein modulations in STAT5A silenced cells were evaluated in western blot analysis and calculated relative to controls, normalized on the GAPDH loading control and expressed as repression percentages. (B) Migration results are shown as mean±SEM (standard error mean) of the area covered by migrated cells normalized to controls. (**C**) The percentage (%) of dead cells displayed in histograms as mean±SEM was evaluated by TMRM and AnnexinV-FITC stainings in a FACS analysis. In bidimensional plots a representative image of ^High^TMRM-^Low^AnnexinV gate (healthy cells) and ^Low^TMRM-^High^AnnexinV gate (dying cells) for each condition is shown. 3 independent biological experiments were performed in triplicate (B) or duplicate (C) and pools or three biological experiments are shown and used for statistics. *P<0.05; **P<0.01; ***P<0.001.

The potential role of STAT5A as a mediator of miR-223 effect on transwell migration or paclitaxel (PTX) induced cell death was evaluated in STAT5A-silenced MDAMB231 cells in which a 40% reduction in protein expression was observed as assessed by western blot analysis (Figure 6A). A 50% decrease was found in cell migration, measured in a transwell assay (Figure 6B). In line with these results, we observed increased transwell migration when STAT5A was overexpressed in MDAMB231 cells (data not shown). A 10–15% increase in PTX-induced cell death (Figure 6C) was observed when cell survival was evaluated by Annexin V-FITC and TMRM staining in FACS analysis following 48h of PTX treatment. These data correlate the role of STAT5A with miR-223 in cell movement and death.

## Discussion

In this work we identified 6 microRNAs enriched in RNA targets among genes differentially expressed in relapsing breast cancer patients. Experimentally, we focused on miR-223 and analyzed its role in cell death induced by chemotherapy compounds and cell migration. We identified STAT5A as direct target of miR-223 and correlated STAT5A with miR-223 functions.

MicroRNAs are well known to play a role in cancer progression [56]. Despite the increasing interest to unravel their role in tumor progression, few miRnomic screenings are available. Here, we attempted microRNA alteration predictions starting from different protein-coding gene profiling of breast cancers using various prediction algorithms and further confirmed the results employing another target reverse gene expression approach [47]. In this way we identified miR-19ab, miR-200bc, miR-203, miR-21, miR-223 and miR-340 as putative players of breast cancer progression. Relevantly, more datasets were used to better represent breast cancer complexity while more algorithms were applied to reduce false positive predictions as shown in [57]. The six predicted microRNAs were already known to be involved in tumor progression [28], [58], [59], [60], [61], [62] and to be poorly expressed in normal breast [63] while expressed in tumors, although diversely in the various subtypes [13], [14], [63]. In particular, miR-19 was reported to be upregulated in the Basal subtypes, miR-200c downregulated in Normal-like tumors and miR-223 downmodulated in luminal-B breast cancers [64]. Moreover miR-223 was found differentially expressed in ER+ and ER- tumors [13], [46], [65]. Subtype-dependent microRNA expression could explain why it is hard to observe differential microRNA expression in total, often unbalanced datasets, by analyzing tumor prognosis (positive or negative relapse). Since our microRNA predictions originated from down-regulated protein-coding genes in tumors with bad prognosis, one could expect an upregulation of the putative microRNAs in Relapse positive versus Relapse negative tumors. However, from our analyses and from similar investigations [13], [64] anti-correlations between microRNAs and targets do not always occur, considering gene expression for tumor samples or cell lines. This could be related to specific features of each microRNA or to the biological systems considered. In addition, circuits with feedback loops involving targets and microRNAs [66] are present in cells, leading to unexpected correlation patterns between the expression levels of microRNAs and their targets.

Clearly our approach is not free of limitations. For sure, we did not predict all the microRNAs involved in breast cancer progression due to the datasets and methodology we used and it is not possible to speculate on their biological role without functional data obtained from cell cultures. Regarding each microRNA function, we are now investigating it in cells and here we present some data relative to miR-223. Considering that our expression analyses revealed that miR-223 is not present in tumor cell lines in culture, while it is expressed in stroma cells, we hypothesized a possible tumor-stroma interaction within the tumor mass. This is supported from data in the literature showing that miR-223 can be transferred to breast tumor cells from bone marrow stroma [67] or microvesicles derived from IL-4 activated macrophages [17]. In addition, the analysis of RNA from punches of tumor or stroma components of breast samples revealed that miR-223 is equally present in the two tumor portions. We further confirmed that conditioned medium (CM) from miR-223 overexpressing fibroblasts or HEK293 cells led to increased miR-223 expression in MDAMB231 cells suggesting a transfer of miR-223 from cell to cell. We exclude an induction of endogenous miR-223 in MDAMB231 cells by secreted factors present in the CM (i.e. growth factors, cytokines), since CM from miR-223-empty cells does not affect miR-223 expression in MDAMB231 cells. Biological analyses on miR-223 overexpressing cells in culture, following CM treatment or overexpression (pre-miR or expression vectors), proved that miR-223 participates to relevant cell functions. No effect was found on cell adhesion and proliferation however a relevant inhibitory role was observed for miR-223 on migration and invasion as well as on cell survival in anoikis conditions or in presence of chemotherapeutic drugs suggesting various interventions during tumor progression. Other microRNAs have been shown to modulate malignancy. For instance, miR-31 and miR-148b control several steps of metastatization from anoikis to invasion and colonization [31], [46]. The function of miR-223 in tumors remains however still unclear and it depends on the kind of analyzed tumor. miR-223 has an anti-proliferative function in cervical and colon-rectal cancer through the targeting of IGFR and FOXO1 [68], [69] and it exerts an anti-metastatic role in oesophageal carcinoma [70]. Instead, it increases proliferation and invasion in gastric cancer [71]. In breast cancer, our findings suggest a suppressive role for miR-223 in tumor progression, similar to what proposed by [67] and more recently by Gong and colleagues [72]. Relevantly, miR-223, like other microRNAs such as miR-31 [73], miR-148b [46] and miR-200bc [74], is involved in drug sensitivity, suggesting a potential function as adjuvant therapy, as recently reported also by [75], [76]. It is important to note that overlapping functions of miR-223 and miR-148b could be due to common target genes, in fact miR-148b shares four nucleotides of the seed region with miR-223.

By using the Ingenuity Pathway Analysis (IPA) for the predicted miR-223 targets, the involvement of miR-223 in cancer and mainly in cell death emerged. Specific enrichment was found for the already validated miR-223 targets, IGFR1 and E2F pro-survival genes and for NRAS, ITGA3 and STAT-family members. Due to the established role of NRAS, ITGA3 and STAT5 in cancer progression and cell death/survival, we focused on them. They were all expressed in breast cancer datasets used for the analysis but only STAT5A was consistently differentially expressed in the datasets used for prediction analysis. miR-223 over-expression was able to downmodulate NRAS, ITGA3 and STAT5 expression at the protein level. In line with miR-223 function are the evidences that integrins, in particular ITGA3 and ITGB1, are key mediators of the outside-in and inside-out signalling in cancer and their depletion leads to decreased migratory abilities and inhibition of metastasis formation [77]. Importantly, they are exploited as possible anti-breast cancer targets [77]. Instead, NRAS is a well-known oncogene, often constitutively active in breast cancer, along with PI3K members and regulators, which are also miR-223 predicted targets. Cells with altered NRAS fail to respond to normal chemotherapeutic treatments and its down-modulation is pursued with different approaches to increase chemotherapy efficacy [78]. STAT5s are transcription factors whose activation needs to be tightly controlled for mammary gland development, lactation and involution [79] and some microRNAs (i.e. miR-222) have been recently found to control STAT5 expression [80]. They are downstream players and crosstalk points of many extracellular signals activated in response to interleukins and growth factors [81]. In non-invasive breast cancer cell lines, STAT5 activation was reported to increase colony formation, invasion and migration via the AKT signalling [82]; while in T47D its activation increases chemotherapy resistance [83]. STAT5A silencing instead leads to better chemotherapy response in leukemia [84], [85]. All these findings support our data, regarding the involvement of miR-223 and its target STAT5A in both anti-migratory and pro-chemotherapeutic effects and guide us to configure miR-223 as a player of the microenvironment in breast cancer. However, even if we proved that STAT5A is a direct target for miR-223 with the luciferase assay, and observed that decreased (RNAi) or increased (cDNA, data not shown) levels of STAT5A lead to modulation of cell migration or chemotherapy induced cell death, further rescuing experiments are necessary to confirm that STAT5A is one of the main players of miR-223. So far we only evidenced a functional correlation between miR223 and STAT5A. Other putative miR-223 targets are currently under investigation.

In conclusion, we identified 6 microRNAs with a role in breast cancer progression and unravelled some functions of miR-223, a small RNA present in tumor and stroma cells, in breast cancer samples. In the future, we will investigate the transfer mechanism of miR-223 from stroma to tumor cells.

## Supporting Information

Figure S1 **miR-223 expression in stroma or tumor cell areas of paraffin-embedded tumor samples. (A)** Top-left: a representative Hematoxilin&Eosin stained section of a paraffin-embedded breast cancer sample. Punches were performed in the embedded tumor in areas corresponding to stroma or tumor cells as indicated by the circles. Magnification of tumor or stoma punches are shown in the top-right or bottom. Levels of magnifications are indicated. **(B)** Relative miR-21 and miR-223 levels of MDAMB231 cells line (reference) or of an infiltrated lymph node (LN, control) or of stroma or tumor cell areas of punches made in blocks of paraffin-embedded infiltrating ductal carcinomas, as measured by qRT-PCR. Two punches for each area of the sample were performed and pooled together for qRT-PCR analyses. Four different blocks were used for stroma or tumor area evaluation. Results are presented as fold changes (mean±SD) relative to miR-223 level in MDAMB231 cells. The delta CT mean of three technical replicates of one (LN) or four (Stroma or Tumors) biological samples were used for statistics. Delta CTs were obtained after normalization on U6sno RNA level. SD = standard deviation; CT = threshold cycle number.
(TIF)

Figure S2 **miR-223 expression levels in various cell lines. (A–F)** Relative miR-223 levels in Mouse Embryo Fibroblasts (MEFs) **(A)** or HEK293 **(B)** or MDAMB231 **(C–E)** or SUM149PT **(F)** cells wild type or previously transduced with pLemiR empty (empty) or miR-223 overexpressing (miR-223) vectors (A–C) or transfected with miR-223 precursors or their negative controls (pre-miR-223 or pre-control) (D, F) or treated with miR-223 overexpressing or control HEK293 (HEK) conditioned medium (CM) (E). Results are presented as fold changes (mean±SD) relative to controls of three technical replicates of one representative biological sample. At least three biological samples were analyzed. Delta CTs were obtained after normalization on U6sno RNA level. SD = standard deviation. *P<0.05; **P<0.01; ***P<0.001.
(TIF)

Figure S3 **Representative images of migration and invasion experiments for miR-223.** Representative images of transwell migration **(top)** or matrigel invasion **(bottom)** assays corresponding to Fig. 3. MDAMB231 cells were transfected with miR-223 or unrelated miR precursors or their negative controls (pre-miR-223 or unrelated pre-miR or pre-control) or stably transduced with pLemiR empty (empty) or miR-223 overexpression (miR-223) vectors or pre-treated for 48 h with conditioned medium (CM) collected from stably transduced HEK293 (HEK) cells (CM HEK empty or CM HEK miR-223).
(TIF)

Figure S4 **Representative images of FACS analysis plots for cell death evaluation.** Referring to Fig. 4, representative images of bidimensional plots of ^High^TMRM-^Low^AnnexinV gate (healthy cells) and ^Low^TMRM-^High^AnnexinV gate (dying cells) of MDAMB231 cells for anoikis experiments **(A)** or Doxorubicin (DOXO) **(B)** or Paclitaxel (PTX) treatments, in presence or absence of ZVAD **(C–E)**. Cells were transiently transfected with miR-223 or with unrelated miR precursors or their negative controls (pre-miR-223 or unrelated pre-miR or pre-control). Alternatively MDAMB231 cells were grown for 48 h in condition medium (CM) collected from HEK293 (HEK) cells stably transduced with pLemiR empty (empty) or miR-223 overexpression (miR-223) vectors and further transferred to regular medium without (Basal) or with PTX for 48 hours and cell death was analyzed **(D).** For Annexin-APC stained cells **(E)** a further gate of ^Low^TMRM-^Low^AnnexinV cells was revealed. Therefore, an additional plot showing the percentage (%) of viable cells after Annexin-FITC Propidium Iodide (PI) staining is presented in **(F)**. ^Low^PI-^Low^AnnexinV gate was reported in the histogram as % of the total cell number. Two independent biological experiments were performed in duplicate and a representative one is shown. In **(F)** duplicates are used for statistics. *P<0.05; **P<0.01; ***P<0.001.
(TIF)
