# Supplementary figures and images for: miR-223 Is a Coordinator of Breast Cancer Progression as Revealed by Bioinformatics Predictions
Source: PLoS One. 2014 Jan 6;9(1):e84859. doi: 10.1371/journal.pone.0084859 (PMC3882278; doi:10.1371/journal.pone.0084859)

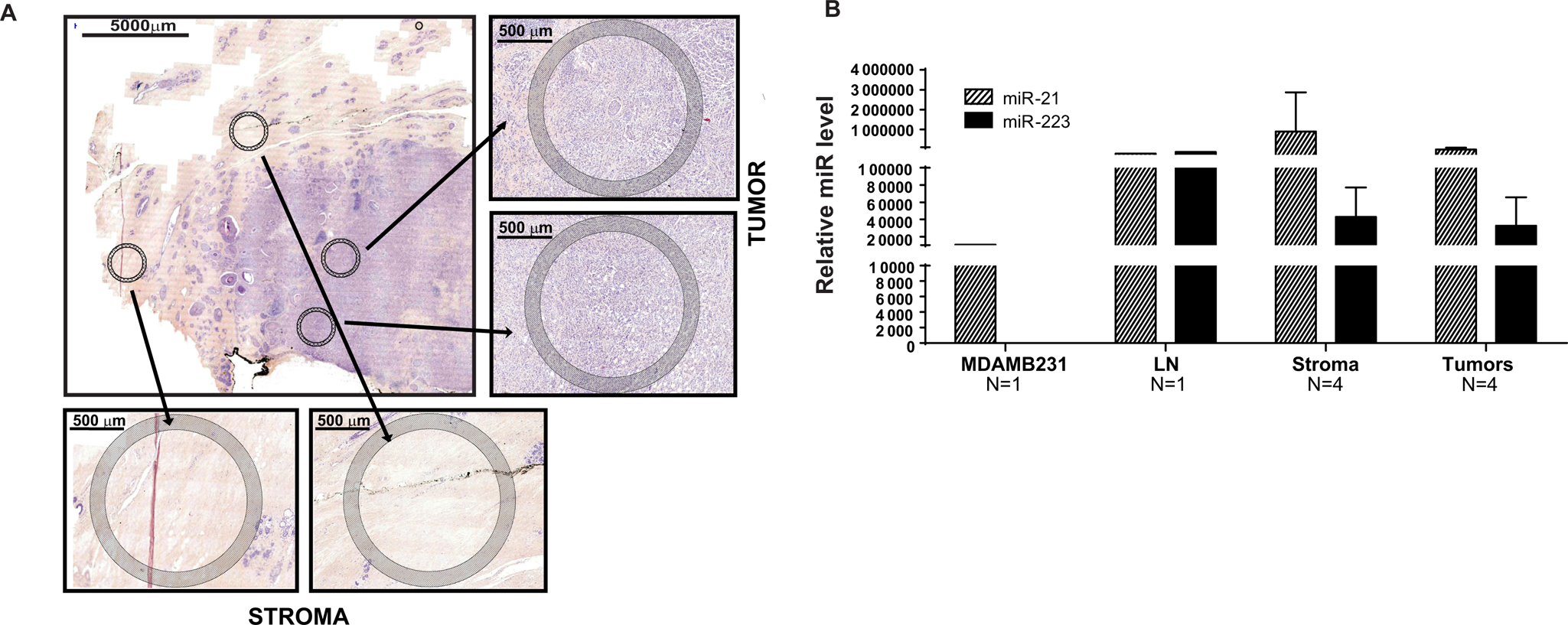

Supplement: Figure S1 — miR-223 expression in stroma or tumor cell areas of paraffin-embedded tumor samples. (A) Top-left: a representative Hematoxilin&Eosin stained section of a paraffin-embedded breast cancer sample. Punches were performed in the embedded tumor in areas corresponding to stroma or tumor cells as indicated by the circles. Magnification of tumor or stoma punches are shown in the top-right or bottom. Levels of magnifications are indicated. (B) Relative miR-21 and miR-223 levels of MDAMB231 cells line (reference) or of an infiltrated lymph node (LN, control) or of stroma or tumor cell areas of punches made in blocks of paraffin-embedded infiltrating ductal carcinomas, as measured by qRT-PCR. Two punches for each area of the sample were performed and pooled together for qRT-PCR analyses. Four different blocks were used for stroma or tumor area evaluation. Results are presented as fold changes (mean±SD) relative to miR-223 level in MDAMB231 cells. The delta CT mean of three technical replicates of one (LN) or four (Stroma or Tumors) biological samples were used for statistics. Delta CTs were obtained after normalization on U6sno RNA level. SD = standard deviation; CT = threshold cycle number. (TIF) [file pone.0084859.s001.tif]

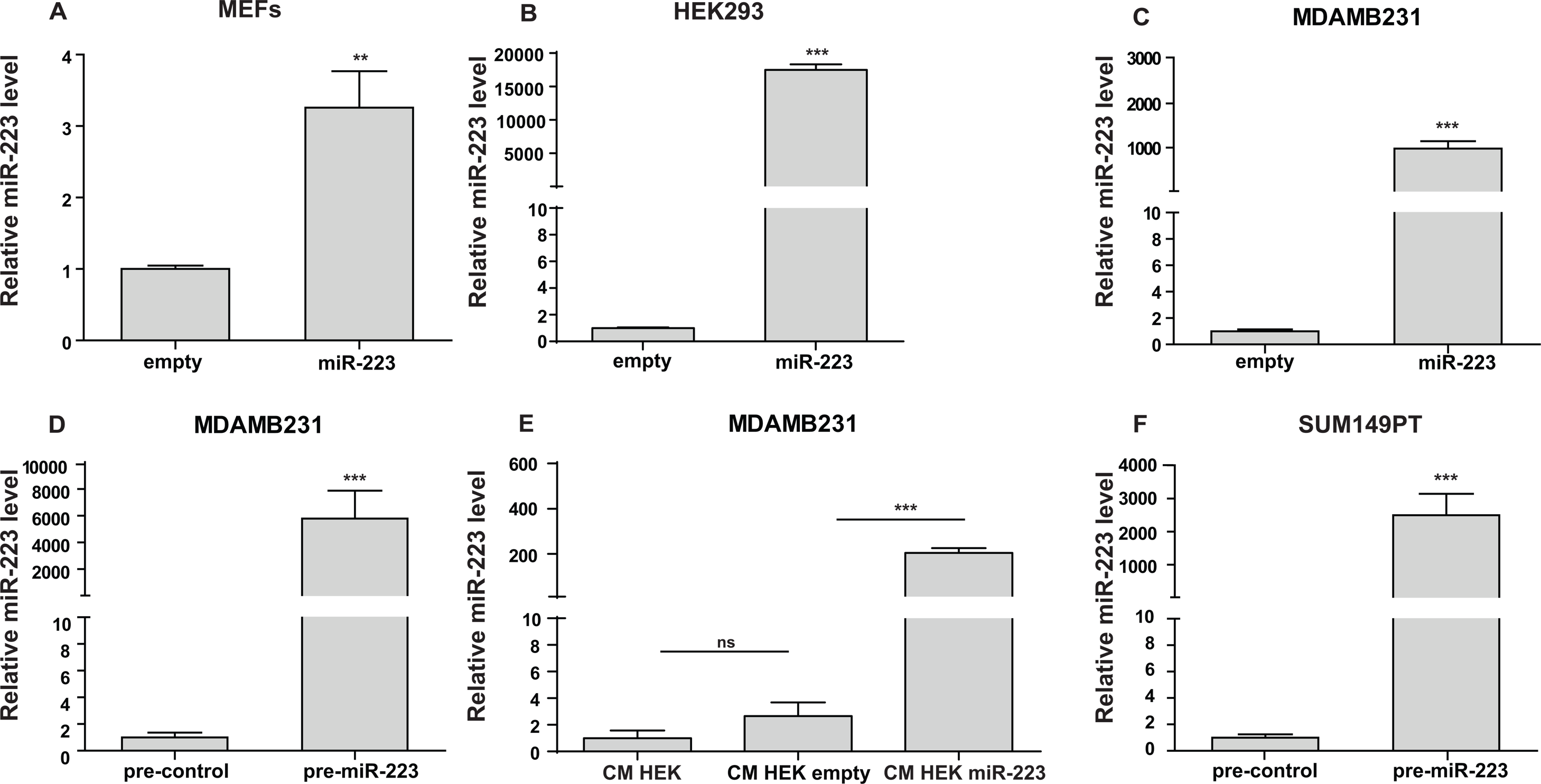

Supplement: Figure S2 — miR-223 expression levels in various cell lines. (A–F) Relative miR-223 levels in Mouse Embryo Fibroblasts (MEFs) (A) or HEK293 (B) or MDAMB231 (C–E) or SUM149PT (F) cells wild type or previously transduced with pLemiR empty (empty) or miR-223 overexpressing (miR-223) vectors (A–C) or transfected with miR-223 precursors or their negative controls (pre-miR-223 or pre-control) (D, F) or treated with miR-223 overexpressing or control HEK293 (HEK) conditioned medium (CM) (E). Results are presented as fold changes (mean±SD) relative to controls of three technical replicates of one representative biological sample. At least three biological samples were analyzed. Delta CTs were obtained after normalization on U6sno RNA level. SD = standard deviation. *P<0.05; **P<0.01; ***P<0.001. (TIF) [file pone.0084859.s002.tif]

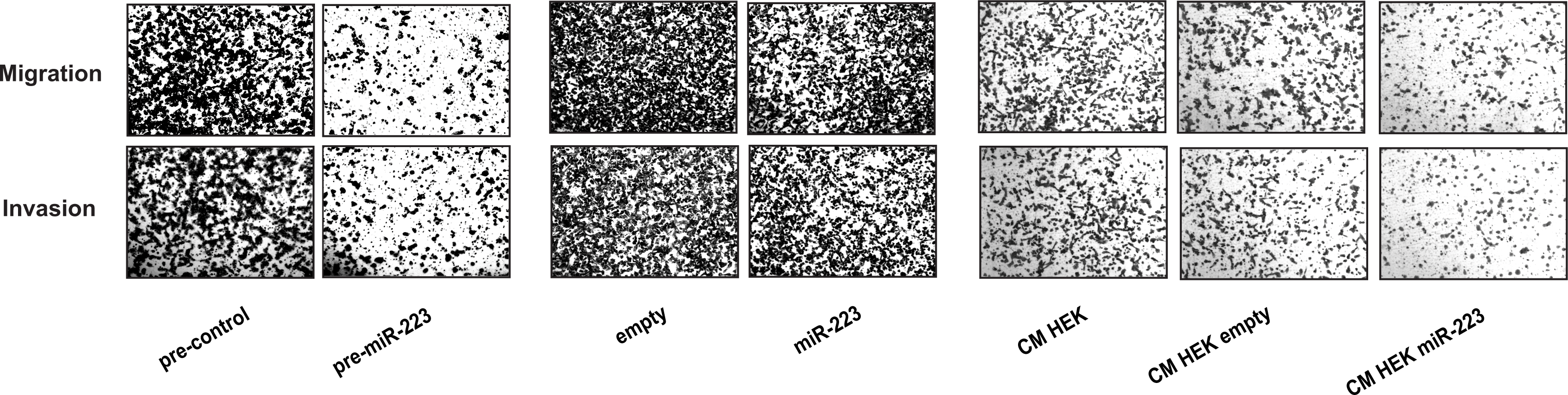

Supplement: Figure S3 — Representative images of migration and invasion experiments for miR-223. Representative images of transwell migration (top) or matrigel invasion (bottom) assays corresponding to Fig. 3. MDAMB231 cells were transfected with miR-223 or unrelated miR precursors or their negative controls (pre-miR-223 or unrelated pre-miR or pre-control) or stably transduced with pLemiR empty (empty) or miR-223 overexpression (miR-223) vectors or pre-treated for 48 h with conditioned medium (CM) collected from stably transduced HEK293 (HEK) cells (CM HEK empty or CM HEK miR-223). (TIF) [file pone.0084859.s003.tif]

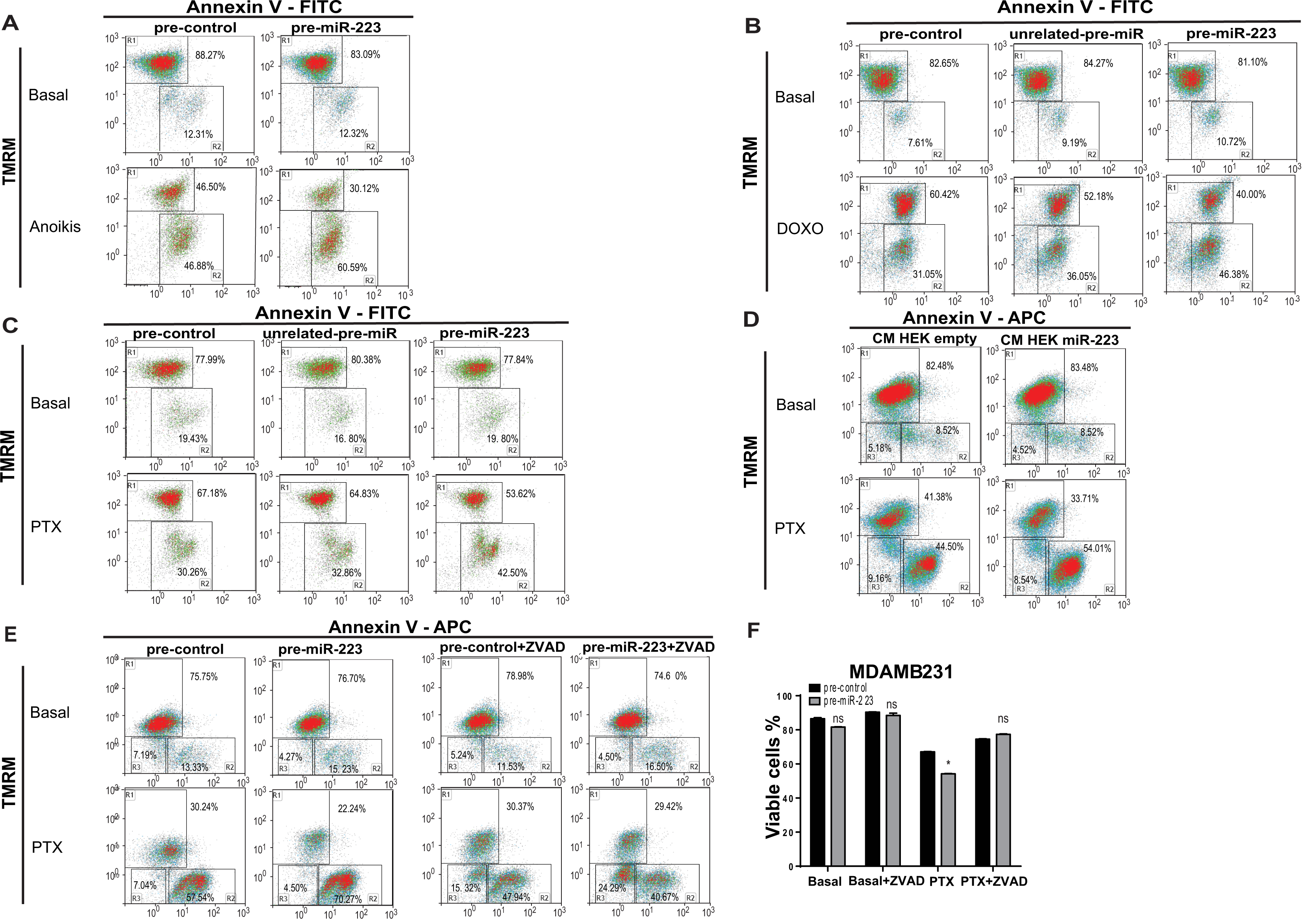

Supplement: Figure S4 — Representative images of FACS analysis plots for cell death evaluation. Referring to Fig. 4, representative images of bidimensional plots of HighTMRM-LowAnnexinV gate (healthy cells) and LowTMRM-HighAnnexinV gate (dying cells) of MDAMB231 cells for anoikis experiments (A) or Doxorubicin (DOXO) (B) or Paclitaxel (PTX) treatments, in presence or absence of ZVAD (C–E). Cells were transiently transfected with miR-223 or with unrelated miR precursors or their negative controls (pre-miR-223 or unrelated pre-miR or pre-control). Alternatively MDAMB231 cells were grown for 48 h in condition medium (CM) collected from HEK293 (HEK) cells stably transduced with pLemiR empty (empty) or miR-223 overexpression (miR-223) vectors and further transferred to regular medium without (Basal) or with PTX for 48 hours and cell death was analyzed (D). For Annexin-APC stained cells (E) a further gate of LowTMRM-LowAnnexinV cells was revealed. Therefore, an additional plot showing the percentage (%) of viable cells after Annexin-FITC Propidium Iodide (PI) staining is presented in (F). LowPI-LowAnnexinV gate was reported in the histogram as % of the total cell number. Two independent biological experiments were performed in duplicate and a representative one is shown. In (F) duplicates are used for statistics. *P<0.05; **P<0.01; ***P<0.001. (TIF) [file pone.0084859.s004.tif]
